# Pan-cancer analysis of prognostic and immunological role of IL4I1 in human tumors: a bulk omics research and single cell sequencing validation

**DOI:** 10.1007/s12672-024-01000-5

**Published:** 2024-05-01

**Authors:** Bin Chen, Yi Liu, Yuping He, Chenfu Shen

**Affiliations:** 1 Department of Radiology, Xiangya Hospital, Central South University, Changsha, Hunan China; 2 Emergency Department, West China Hospital, Sichuan University, Chengdu, Sichuan China; 3 Health Management Center, Xiangya Hospital, Central South University, Changsha, Hunan China; 4 Department of Neurosurgery, Xiangya Hospital, Central South University, Changsha, Hunan China; 5 National Clinical Research Center for Geriatric Disorders, Xiangya Hospital, Central South University, Changsha, Hunan China

**Keywords:** Interleukin-4 inducible gene 1, Pan-cancer, Tumor immune microenvironment, Tumor immunosuppressive status, Tumor-associated macrophages

## Abstract

**Background:**

Interleukin-4 inducible gene 1 (IL4I1) regulates tumor progression in numerous tumor types. However, its correlation with immune infiltration and prognosis of patients in a pan-cancer setting remains unclear.

**Methods:**

Data from the Cancer Genome Atlas (TCGA), Genotype-Tissue Expression (GTEx), UALCAN, Clinical Proteomic Tumor Analysis Consortium (CPTAC), Gene Expression Omnibus (GEO), cBioPortal, Cancer Single-cell State Atlas (CancerSEA), and Tumor IMmune Estimation Resource(TIMER) databases were used to evaluate IL4I1 expression, clinical features and prognostic effects, gene set enrichment, and correlation with immune cell infiltration, as well as the relationship between IL4I1 methylation and expression and survival prognosis. Correlations with 192 anticancer drugs were also analyzed.

**Results:**

IL4I1 was significantly overexpressed in the majority of tumors, and the imbalance of IL4I1 was significantly correlated with overall survival and pathological stage. Moreover, total IL4I1 protein was increased in cancer. Therefore, IL4I1 may be used as a prognostic biomarker or protective factor in numerous types of cancer. The methylation level of IL4I1 may also be used as a prognostic marker. The functional enrichment of IL4I1 was closely related to the immunomodulatory pathway. In addition, the level of tumor-associated macrophage infiltration was positively correlated with the expression of IL4I1 in pan-cancerous tissues. scRNA-seq analysis suggested that IL4I1 differ significantly among different cells in the tumor microenvironment and was most enriched in macrophages. Various immune checkpoint genes were positively correlated with IL4I1 expression in most tumors. In addition, patients with high IL4I1 expression may be resistant to BMS-754807 and docetaxel, but sensitive to temozolomide.

**Conclusion:**

IL4I1 may play a role as promoter of cancer and prognostic indicator in patients. High expression of IL4I1 is associated with the state of tumor immunosuppression and may contribute to tumor-associated macrophage invasion. Therefore, IL4I1 may be a new therapeutic target for the treatment and prognosis of patients with cancer.

**Supplementary Information:**

The online version contains supplementary material available at 10.1007/s12672-024-01000-5.

## Introduction

Interleukin-4 inducible gene 1 (IL4I1), a member of the flavin adenine dinucleotide (FAD)-binding enzyme family, functions as an L-amino acid oxidase (LAAO). It was first described in B cells and subsequently detected in dendritic cells and tumor-associated macrophages (TAM) [1]. By catalyzing the oxidation of phenylalanine into phenylpyruvate and hydrogen peroxide, IL4I1 inhibits T cell proliferation. H_2_O_2_ is responsible for the transient downregulation of T-cell receptor expression [2]. IL4I1 is also expressed by a number of T cell subsets, including T helper 17 (Th17) and regulatory T (Treg) cells [2, 3]. Controlled by retinoic-acid-receptor-related orphan nuclear receptor gamma (RoRγ) T, its transcriptional expression in CD4 + T cells is evident in Th17 and T cells differentiating from immature T or Treg cells [4]. Recent data showed that different types of human cancers directly produce IL4I1 to form metabolites that activate aromatic receptors (aryl hydrocarbon receptor [AhR]). Besides phenylalanine, IL4I1 can decompose tyrosine and tryptophan, producing hydroxyphenylpyruvate and indole-3-pyruvate. This activation of AhR facilitates cancer cell movement and inhibits the immune response[5].

Previous studies have shown that IL4I1 is associated with adverse outcomes in some human cancers (e.g., breast cancer, renal cell carcinoma, and glioma) [6–8]. Sadik et al. recently found IL4I1 expression in tumor cells of low-grade gliomas and glioblastomas, and observed a correlation with reduced patient survival [9]. Mazzoni et al. reported that mesenchymal stromal cells derived from head and neck cancer express IL4I1 and participate in the inhibition of T cell proliferation [10]. Therefore, IL4I1 may be abnormally expressed in tumors, thus affecting the development of tumors. However, currently, there is no comprehensive study on the role of IL4I1 in the pan-cancer setting.

A number of studies have shown that the tumor immune microenvironment (TIME) has clinicopathological significance in predicting the therapeutic effect and prognosis of patients with cancer [11, 12]. It has been confirmed that solid tumors are composed of malignant, non-malignant, hematopoietic, and mesenchymal cells. Among non-malignant cells, TAM play an important role in promoting tumor progression [13, 14]. High levels of TAM in cancerous tissue can affect the immune escape state of the tumor, resulting in ineffective immunotherapy [15]. Therefore, exploring the relationship between IL4I1 and TAM is helpful in understanding the role of IL4I1 in tumor progression.

In this study, we investigated the expression of IL4I1 and its relationship with the prognosis of patients with cancer. We further studied the relationship between IL4I1 and the immune cell infiltration score, chemokines and chemokine receptors, major histocompatibility complex (MHC), immune activation genes, immunosuppressive genes, and immune checkpoints. Our results provide new insights into the functional role of IL4I1 in the pan-cancer setting and highlight the potential mechanism underlying the effects of IL4I1 on the tumor microenvironment and cancer immunotherapy.

## Material and methods

### Single-cell transcriptome sequencing data analysis

Analysis of IL4I1 mRNA expression in the pan-cancer cohort was conducted using the Sanger Box website (http://sangerbox.com/Tool). Website analysis data from The Cancer Genome Atlas (TCGA) and Genotype-Tissue Expression (GTEx). We used the Clinical Proteomic Tumor Analysis Consortium (CPTAC) dataset from the UALCAN website (http://ualcan.path.uab.edu/analysis-prot.html) to analyze the total protein of IL4I1 between primary and normal tumors. The expression level. ScRNA-seq preparation and data analysis were conducted as previously described [16]. Single-cell sequencing datasets were downloaded from the GEO database (GSE103224). Additionally, Gene Expression Profiling Interactive Analysis 2 (GEPIA2; http://gepia2.cancer-pku.cn/#index) was utilized to analyze the staging map of IL4I1 expression across various pathological stages (stage I–IV) of all TCGA tumors.

### Gene alteration

We studied changes in the IL4I1 gene in the pan-cancer cohort using the cBioPortal portal (https://www.cbioportal.org/) [17]. For this purpose, we analyzed residual changes in “TCGA Pan-Cancer Atlas Studies” in the “Quick select” section by entering the term “IL4I1”. The results are shown in the “Cancer Types Summary” module, including the type of mutation and the results with regard to copy number change. The comparison module was also used to obtain the survival data of patients with cancer from TCGA with or without changes in the IL4I1 gene, namely overall survival (OS), disease-specific survival (DSS), disease-free survival, and progression-free survival.

### Prognostic analysis

Kaplan–Meier analysis was used to evaluate the OS of IL4I1 in TCGA cohort. Univariate Cox regression analysis was used to evaluate the significance of IL4I1 in predicting OS and DSS of patients in the pan-cancer setting.

### IL4I1-binding protein network and methylation analysis

We used the Search Tool for the Retrieval of Interacting Genes (STRING) website (https://string-db.org/), and entered the terms “IL4I1” and “Homo sapiens” to map a network of protein–protein interactions. We used the GSCALite platform (http://bioinfo.life.hust.edu.cn/web/GSCALite/) [18] to analyze the differential expression of IL4I1 and enriched genes, methylation, the correlation between methylation and methylation, and the relationship between methylation and OS in different types of carcinomas and their paired normal tissues from TCGA. After logging on to the GSCALite website, we used the “TCGA Cancer-methylation” module to select 32 TCGA cancer types. Subsequently, we analyzed the differential methylation of IL4I1 and its transcription factors between tumors and normal tissues. In the final map produced by GSCALite, only 14 cancer types showed methylation profiles of IL4I1 and its transcription factors. Thereafter, using the same platform, we further investigated the relationship between methylation and the expression of IL4I1 and enriched genes in different types of cancer.

### Analysis of the relationship between IL4I1 expression and TIME

The TIMER2.0 database (https://cistrome.shinyapps.io/timer/) was used to analyze the relationship between the expression of IL4I1 in pan-cancer tissues and immune cell infiltration scores [19]. We also conducted a correlation analysis between IL4I1 expression and 150 marker genes of five immune pathways, i.e., chemokine (41), receptor (18), MHC (21), immunoinhibitor (24), and immunostimulator (46), in the pan-cancer setting. We completed the correlation analysis using the Sanger Box website (http://sangerbox.com/Tool).

### Cancer single-cell state atlas (CancerSEA)

CancerSEA (http://biocc.hrbmu.edu.cn/CancerSEA/home.jsp) [20] provides functional state mapping of cancer single cells, including 14 functional states (stem cell, invasion, metastasis, proliferation, EMT, angiogenesis, apoptosis, cell cycle, differentiation, DNA damage, DNA repair, hypoxia, inflammation, and quiescence) from 25 cancer types. In the present study, CancerSEA was used to explore the expression profile of LI4I1 at a single-cell level and its potential functional status in pan-cancer.

### Gene set enrichment analysis (GSEA)

The GSEA online database (https://www.gsea-msigdb.org/gsea/downloads.jsp) was used for the Kyoto Encyclopedia of Genes and Genomes (KEGG) analysis. The biological function of IL4I1 in the pan-cancer setting was explored by GSEA analysis, which was performed using the R packet “cluster Profiler” [21].

### Drug sensitivity of IL4I1

We downloaded half maximal inhibitory concentration (IC50) values and gene expression profiles of drugs in related cell lines from the Genomics of Drug Sensitivity in Cancer (GDSC) database (https://www.cancerrxgene.org/).

### Quantitative real-time polymerase chain reaction (qRT-PCR) analysis

In our study, 68 cases of glioma and 15 normal tissues were collected from August 2018 to May 2020 at Xiangya Hospital. All patients underwent surgical resection of glioma or non-tumor operations, such as craniocerebral trauma, which were performed in accordance with the Helsinki Declaration. Detailed clinical information is detailed in Supplementary Table 1. After surgical resection, all specimens were frozen in liquid nitrogen and stored at – 80 °C until RNA was extracted. As mentioned in our previous approach [22], the RNA was extracted from tissues using TRIzol Reagent (Invitrogen, CA, USA) and was reverse transcribed into cDNA using the PrimeScriptRT kit (Takara, Japan). The SYBR Green PCR MasterMix kit (Takara) and ABI7500 system (Applied Biosystems, CA, USA) for qRT-PCR. PCR amplification conditions were: initial denaturation for 30 s at 95 ℃, and 40 cycles at 95 ℃ for 10 s and 60 ℃ for 30 s. GAPDH was used as a control. The following primer sequences were used: IL4I1 Forward: 5′- ACTCGCCCGAAGACATCTAC-3′, Reverse: 5′- CATCCTCGGACATCACGTCTC-3′; GAPDH Forward, 5′-CCAGGTGGTCTCCTCTGA-3′ and Reverse 5′-GCTGTAGCCAAATCGTTGT-3′. IL4I1 expression level was calculated by the 2^−ΔΔCT^ method. The study was approved by the Ethics Committee of Xiangya Hospital of Central South University (no.202009719), and informed consent was obtained from all patients.

### Statistical analysis

The data are expressed as the average ± standard deviation. Differences between groups were analyzed using Student’s *t*-test. The R4.1.2 software was used for statistical analysis. The p-values < 0.05 denoted statistically significant differences.

## Results

### Expression of IL4I1 in the pan-cancer setting

**Fig. 1:**
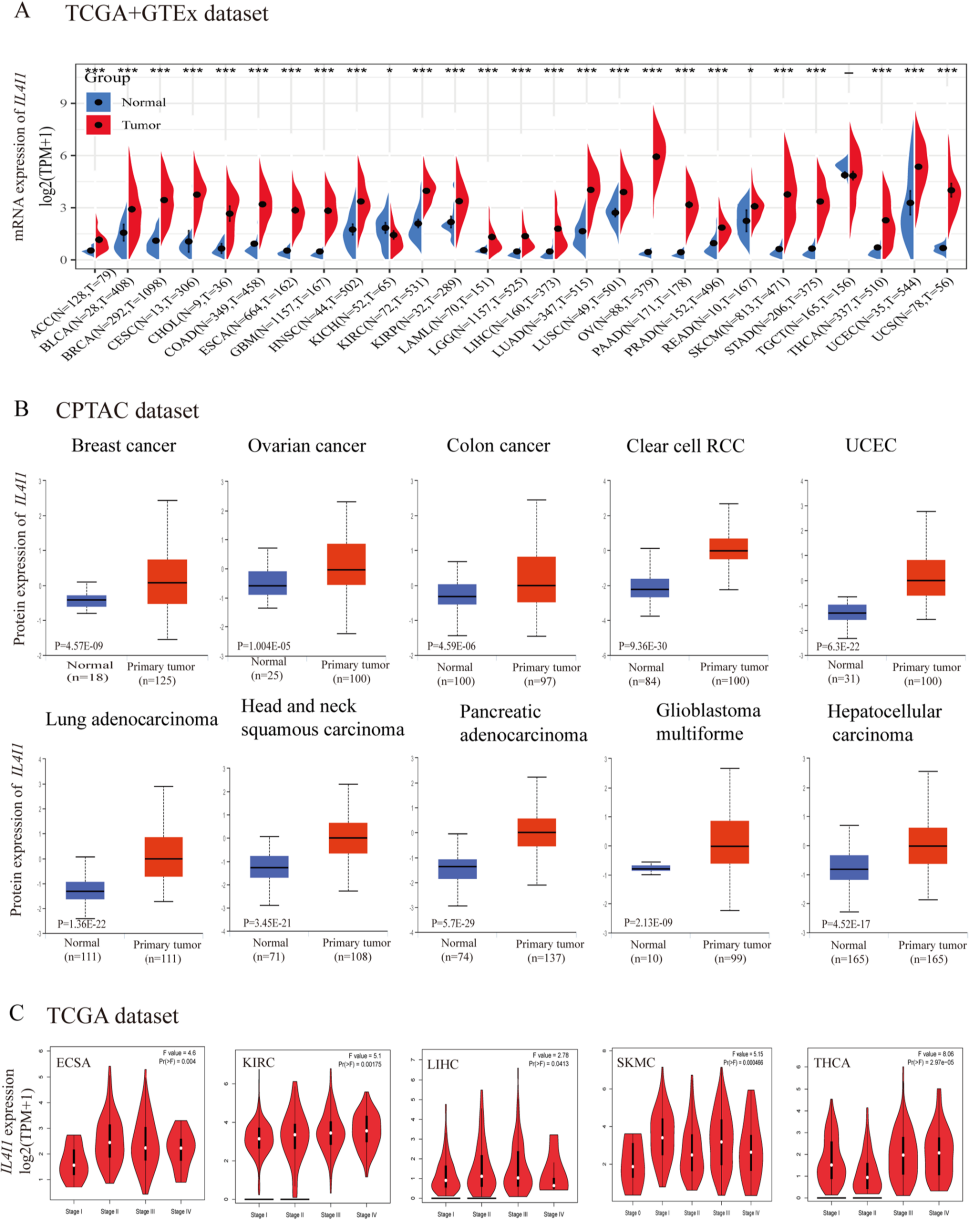
IL4I1 expression. **A** Expression of IL4I1 in tumor and normal tissues in the TCGA + GTEx dataset. **B** IL4I1 total protein in tumor tissues compared with normal tissues. **C** IL4I1 expression in different stages of various cancers (ESCA, KIRC, KICH, LIHC, SKCM, and THCA)

We analyzed the differential expression of IL4I1 in 33 cancers using the TCGA + GTEx database. A significant difference in IL4I1 expression was observed between cancer and normal tissues. The data showed that the expression of IL4I1 was high in 25 types of tumors, namely adrenocortical carcinoma (ACC), bladder urothelial carcinoma, breast invasive carcinoma (BRCA), cervical squamous cell carcinoma and endocervical adenocarcinoma (CESC), cholangiocarcinoma, colon adenocarcinoma (COAD), esophageal carcinoma (ESCA), GBM, HNSC, kidney renal clear cell carcinoma (KIRC), kidney renal papillary cell carcinoma (KIRP), acute myeloid leukemia (LAML), brain lower grade glioma (LGG), liver hepatocellular carcinoma (LIHC), LUAD, lung squamous cell carcinoma (LUSC), ovarian serous cystadenocarcinoma (OV), rectum adenocarcinoma (READ), PRAD, skin cutaneous melanoma (SKCM), stomach adenocarcinoma (STAD), thyroid carcinoma (THCA), UCEC, and uterine carcinosarcoma (UCS) compared with normal tissues; however, the levels of IL4I1 were lower in kidney chromophobe (KICH) (all p < 0.05) (Fig. 1A).

Next, the CPTAC dataset analysis showed that the expression of IL4I1 total protein in BRCA, COAD, OV, KIRC, HNSC, PAAD, GBM, LIHC, UCEC, and LUAD was significantly higher than that recorded in normal tissues (Fig. 1B). Subsequently, the results of the pathological staging map using the GEPIA2 database also showed a significant correlation between the expression of IL4I1 and the pathological stages of ESCA (p = 0.004), KIRC (p = 0.00175), LIHC (p = 0.0413), SKMC (p = 0.000466), and THCA (p = 2.97e-5) (Fig. 1C). In summary, we found that the mRNA and protein expression levels of IL4I1 were significantly high in the vast majority of tumors, and had a significant correlation with the pathological stage.

### Gene alteration

**Fig. 2:**
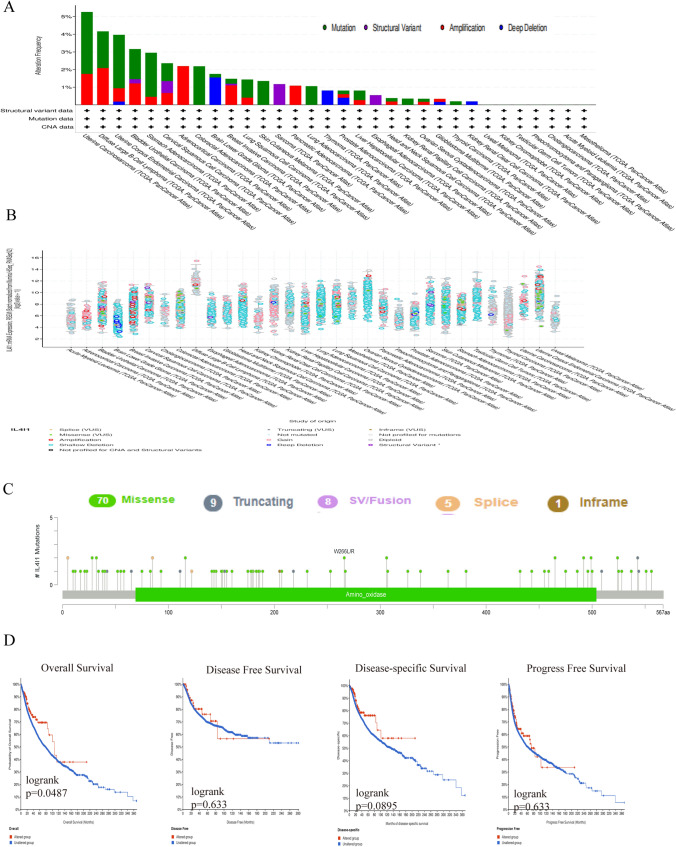
Alterations in the IL4I1 gene. **A** Frequency of IL4I1 mutation in multiple pan-cancer studies from the TCGA using the cBioPortal database. **B** General mutation count for IL4I1 in various cancer types from TCGA using the cBioPortal database. **C** Mutation diagram for IL4I1 in different cancer types across protein domains. **D** OS, DSS, DFS, and PFS of patients with cancers carrying genetic alterations

We further investigated changes in the IL4I1 gene in pan-cancer samples from TCGA using the cBioPortal. The highest frequency of gene changes was found in UCS, diffuse large B-cell lymphoma, and UCEC (5.26%, 4.17%, and 3.97%, respectively) (Fig. 2A, B). A total of 93 mutation sites (70 missense, nine truncation, eight fusion, five splice, and one inframe) were detected between amino acids 0–567 (Fig. 2C). In addition, we studied whether changes in the IL4I1 gene affect the survival and prognosis of patients with tumors. Based on the cBioPortal database analysis, we found statistically significant differences in the OS rate (p = 0.0487); however, there were no statistically significant differences in disease-free survival (p = 0.663), DSS (p = 0.0895), and progression-free survival (p = 0.633) (Fig. 2D). Hence, we concluded that the frequency of change in the IL4I1 gene was low. Consequently, the effect of genetic changes on the prognosis of patients was negligible.

### Prognostic role of IL4I1

**Fig. 3:**
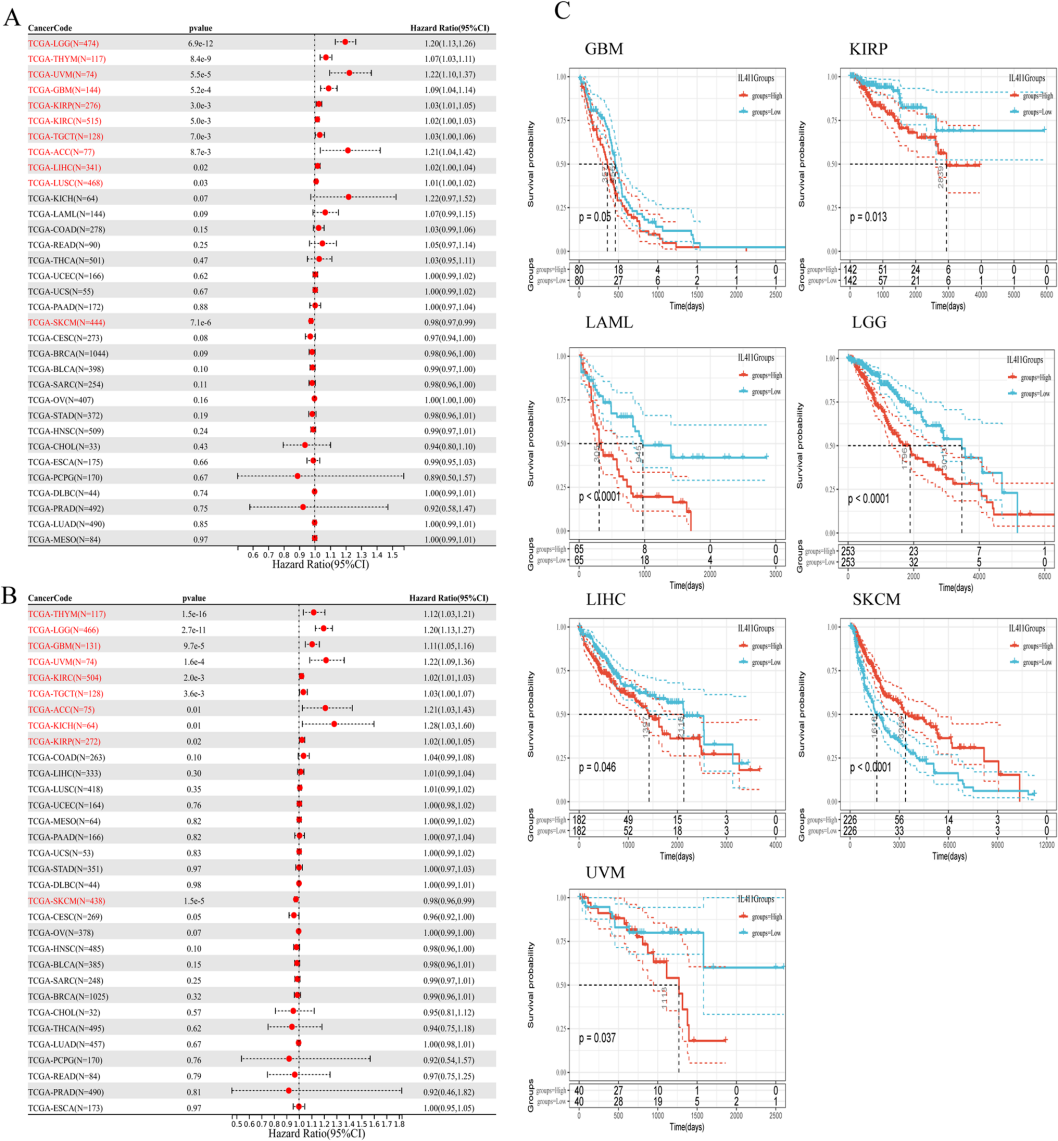
Survival analysis comparing the high and low expression of IL4I1 in different types of cancer in TCGA cohort. **A**, **B** Relationship between IL4I1 expression and prognosis (OS and DSS) of patients with different cancers in TCGA cohort. **C** Survival curves of OS with significance for seven types of cancer (i.e., GBM, KIRP, LAML, LGG, LIHC, SKCM, and UVM) in TCGA

We further evaluated the significance of IL4I1 as a prognostic indicator for patients with tumors. Univariate Cox regression and Kaplan–Meier survival analyses were conducted using TCGA pan-cancer clinical data from the UCSC database. The result table of univariate Cox regression analysis. IL4I1 is a risk factor for OS in patients with LGG (hazard ratio [HR] = 1.20, p = 6.9e-12), THYM (HR = 1.07, p = 8.4e-9), UVM (HR = 1.22, p = 5.5e-5), GBM (HR = 1.09, p = 5.2e-4), KIRP (HR = 1.02, p = 3.0e-3), KIRC (HR = 1.02, p = 5.0e-3), TGCT (HR = 1.03, p = 7.0e-3), ACC (HR = 1.21, p = 8.7e-3), LIHC (HR = 1.02, p = 0.02) and LUSC (HR = 1.01, p = 0.03); nevertheless, it appears to be a protective factor in SKCM (HR = 0.98, p = 7.1e-6) (Fig. 3A). In addition, to avoid deviations caused by non-cancer events, we performed DSS univariate Cox regression analysis. As shown in Fig. 3B, IL4I1 is a risk factor for DSS in patients with THYM (HR = 1.12, p = 1.5e-16), LGG (HR = 1.20, p = 2.7e-11),GBM (HR = 1.11, p = 9.7e-5), UVM (HR = 1.22, p = 1.6e-4), KIRC (HR = 1.02, p = 2.0e-3), TGCT (HR = 1.03, p = 3.6e-3), ACC (HR = 1.21, p = 0.01), KICH (HR = 1.28, p = 0.01), and KIRP (HR = 1.02, p = 0.02). Notably, it is a protective factor in SKCM (HR = 0.98, p = 1.5e-5).

Kaplan–Meier survival analysis showed that high IL4I1 expression predicted worse OS in patients with GBM, KIRP, LAML, LGG, LIHC, and UVM; in contrast, it was associated with better OS in patients with SKCM (Fig. 3C). These results suggest that the high expression of IL4I1 plays a major role in tumorigenesis in most tumor types.

### GSEA of IL4I1

**Fig. 4:**
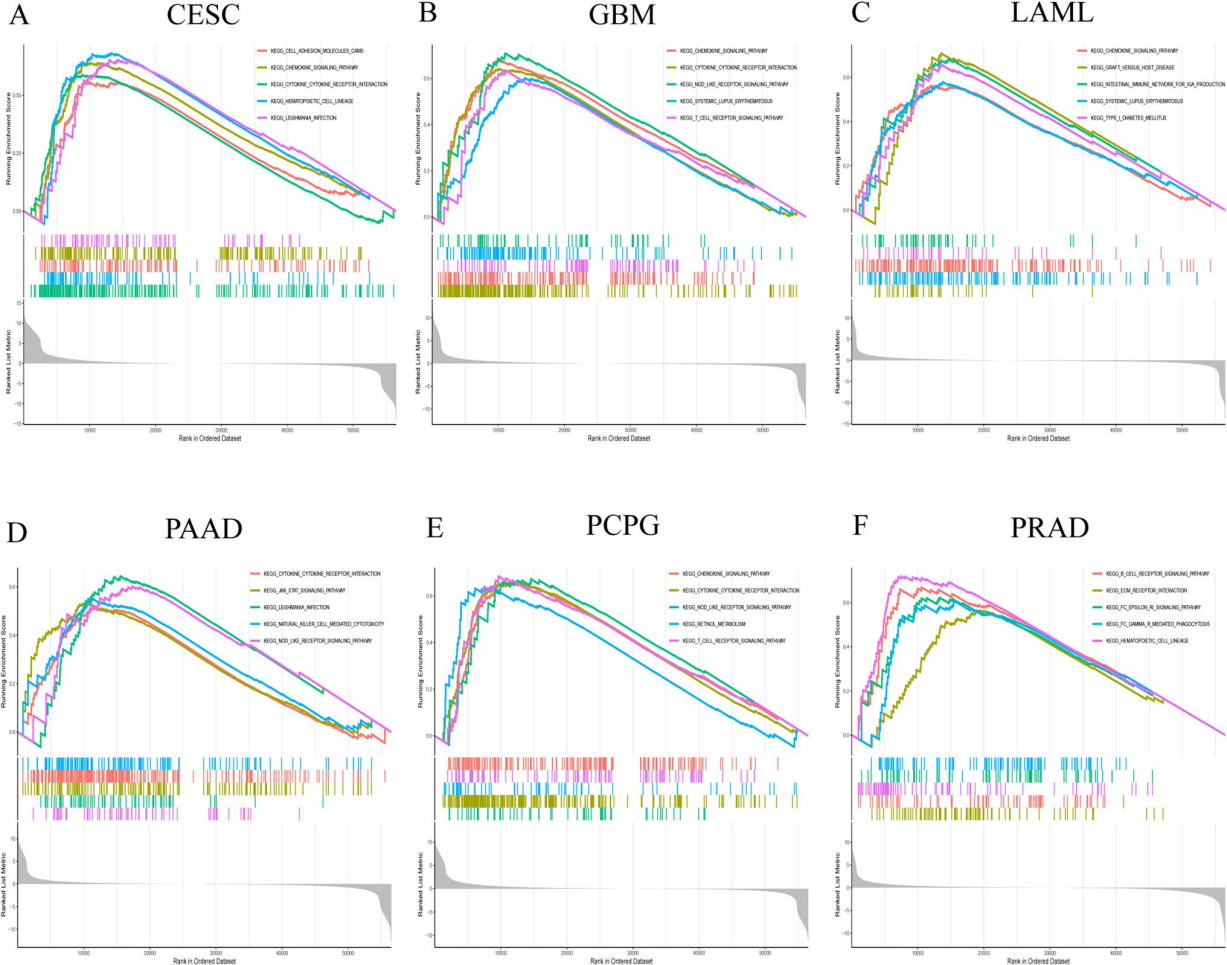
GSEA of IL4I1 in the pan-cancer setting. **A**–**F** The top 5 results of the GSEA in indicated tumor types (NES ≥ 1.5; adjusted p-value < 0.05)

We performed GSEA and KEGG pathway enrichment analysis to evaluate the role of IL4I1 in 33 tumors from TCGA. The results showed that IL4I1 is closely related to immune-related pathways, such as “T cell receptor signaling”, “intestinal immune network for IgA production”, “chemokine signaling”, “cytokine-cytokine receptor interaction” participating in the immunomodulatory effect between lymphoid and non-lymphocytic pathways. This was particularly observed in CESC, GBM, LAML, PPAD, pheochromocytoma and paraganglioma, PRAD, and other tumors (Fig. 4A–F). These results suggest that IL4I1 is closely related to the regulation of TIME and ligand receptor interaction between lymphoid and malignant tumor cells.

### Analysis of immune cell infiltration

**Fig. 5:**
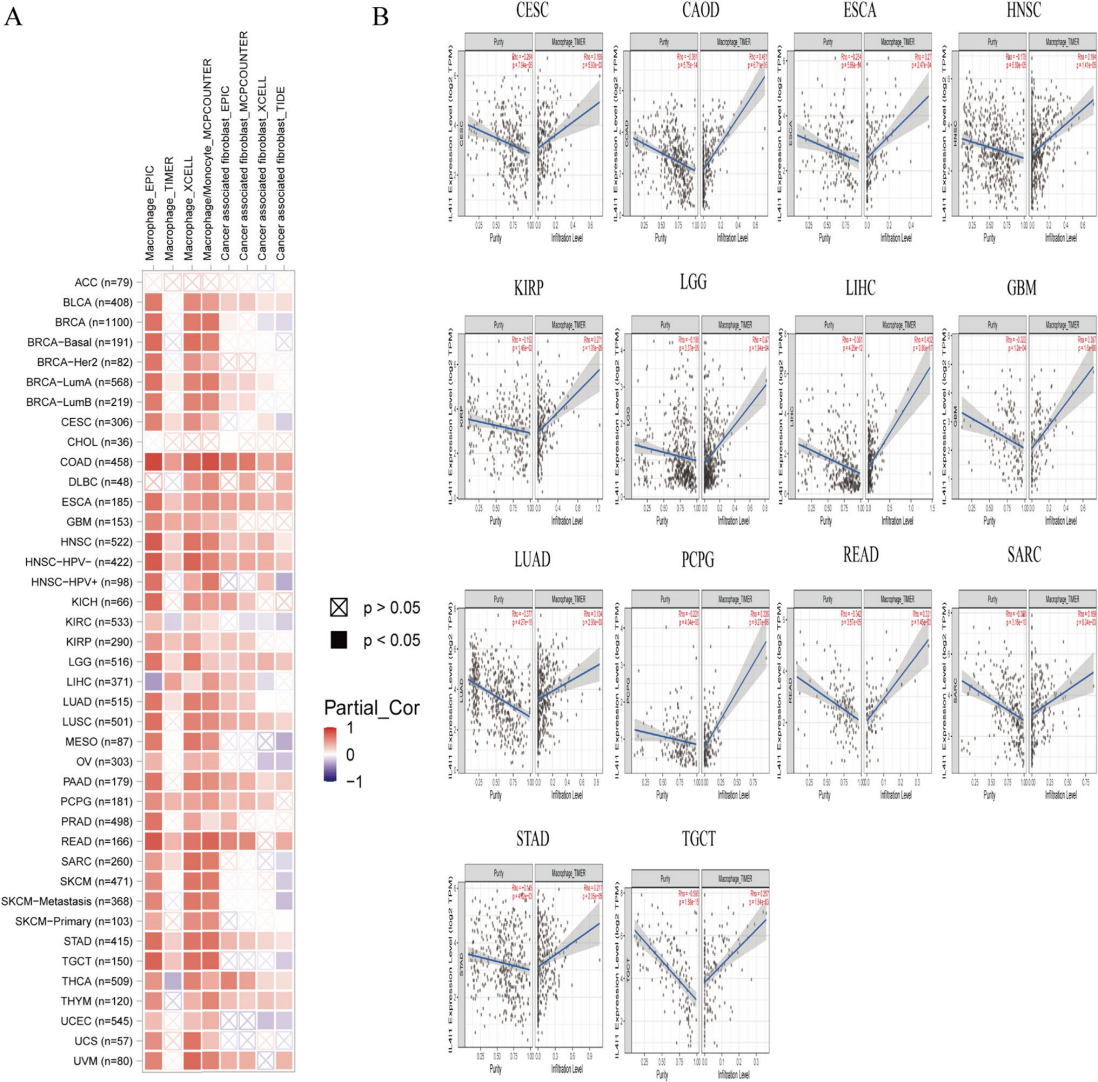
Immune cell infiltration analysis. **A** Correlation between IL4I1 expression and the levels of TAM and CAF infiltration in pan-cancer samples from TCGA using the TIMER2 database. **B** Correlation between IL4I1 expression and tumor purity, and TAM infiltration levels in pan-cancer samples from TCGA using the TIMER2 database

After revealing that IL4I1 is closely related to the immunomodulatory pathway by GSEA analysis, we focused on the invasion of immune cells in the tumor microenvironment. Using the TIMER2 database, we found that the expression of IL4I1 was positively correlated with the degree of invasion of TAM and cancer-associated fibroblasts (CAF) in pan-cancerous tissues (Fig. 5A). Our results also showed that the expression of IL4I1 in CESC, COAD, ESCA, HNSC, KIRP, LGG, LIHC, GBM, LUAD, pheochromocytoma and paraganglioma, READ, sarcoma, STAD, and testicular germ cell tumors was negatively correlated with tumor purity and positively correlated with the degree of TAM invasion (Fig. 5B).

**Fig. 6:**
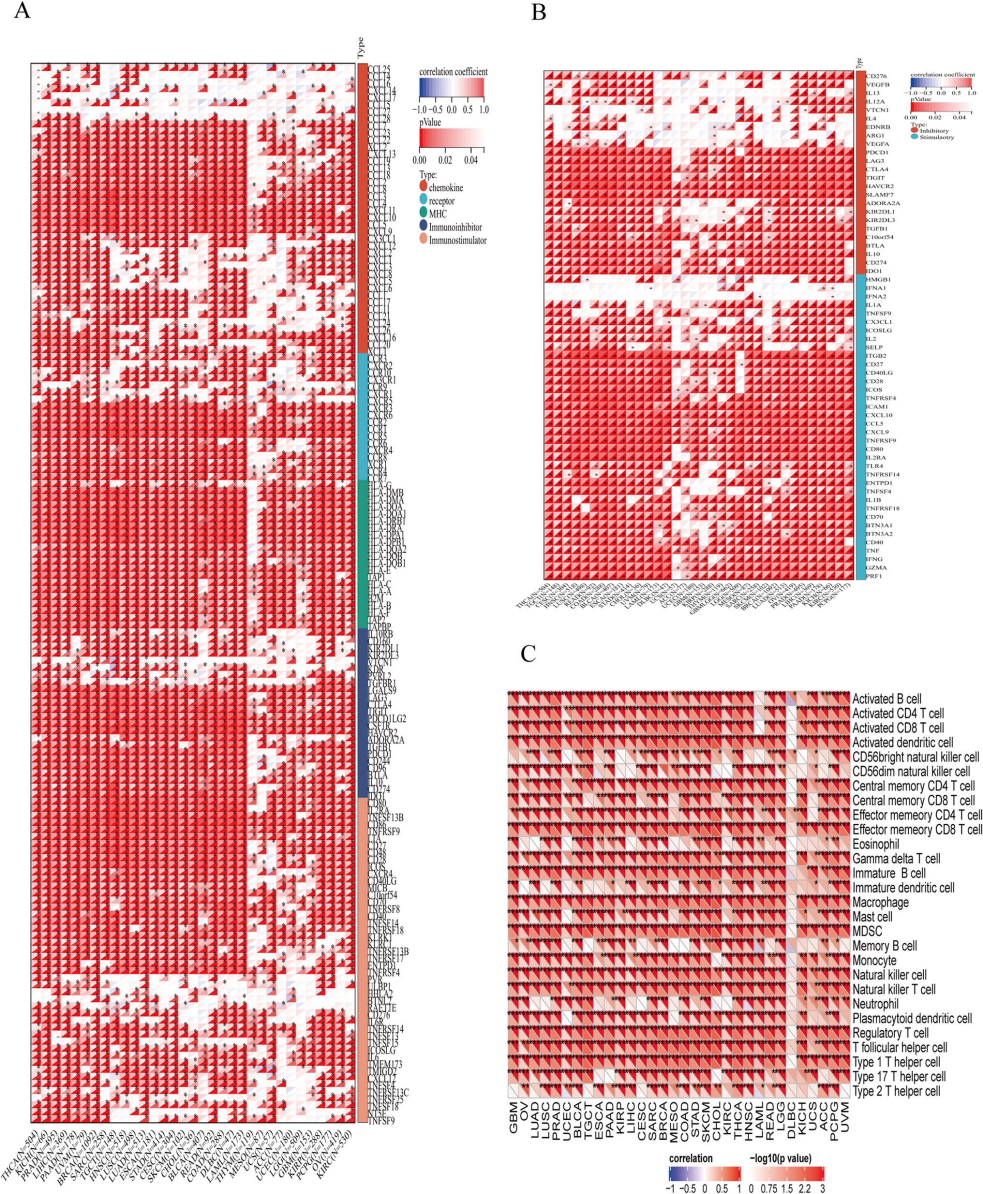
Correlation between IL4I1 and immunosuppressive genes. **A** Heatmap representing the correlation between IL4I1 expression and immune checkpoints, MHC, immunostimulatory genes, immunosuppressive genes, chemokines, and chemokine receptors. **B** Heatmap representing the correlation between IL4I1 expression and chemokine genes. **C** Heatmap representing the correlation between IL4I1 expression and 28 tumor-associated immune cells. The Pearson’s correlation coefficient was calculated using the R software. *p < 0.05, **p < 0.01, ***p < 0.001, and ****p < 0.0001

Next, we examined the relationship between IL4I1 and immune checkpoints, MHC, immunostimulatory genes, immunosuppressive genes, chemokines, and chemokine receptors. The vast majority of chemokines and chemokine receptors, such as C–C motif chemokine ligand 5 (CCL5), C-X-C motif chemokine ligand 9 (CXCL9), CXCL10, C-X-C motif chemokine receptor 6 (CXCR6), C–C motif chemokine receptor 1 (CCR1), and CCR5, were positively correlated with IL4I1 in most tumors. Similarly, MHC, immunostimulatory genes, and immunosuppressive genes, such as major histocompatibility complex, class I, E (HLA-E), HLA-B, hepatitis A virus cellular receptor 2 (HAVCR2), colony stimulating factor 1 receptor (CSF1R), programmed cell death 1 ligand 2 (PDCD1LG2), CD86, CD27, CD28, and TNF receptor superfamily member 9 (TNFRSF9), were positively correlated with IL4I1 in most tumors (Fig. 6A). Furthermore, most immune checkpoint genes, such as cytotoxic T-lymphocyte-associated protein 4 (CTLA4), PDCD1, CD274, CD80, and perforin 1 (PRF1), were positively correlated with IL4I1 in most tumors (Fig. 6B). Using the correlation between IL4I1 and 28 tumor-associated immune cells calculated by a single-sample GSEA algorithm, it was found that IL4I1 was positively correlated with most immune cells, particularly TAM (Fig. 6C).

### Expression profile of IL4I1 in a single-cell level and its potential functional status in pan-cancer

**Fig. 7:**
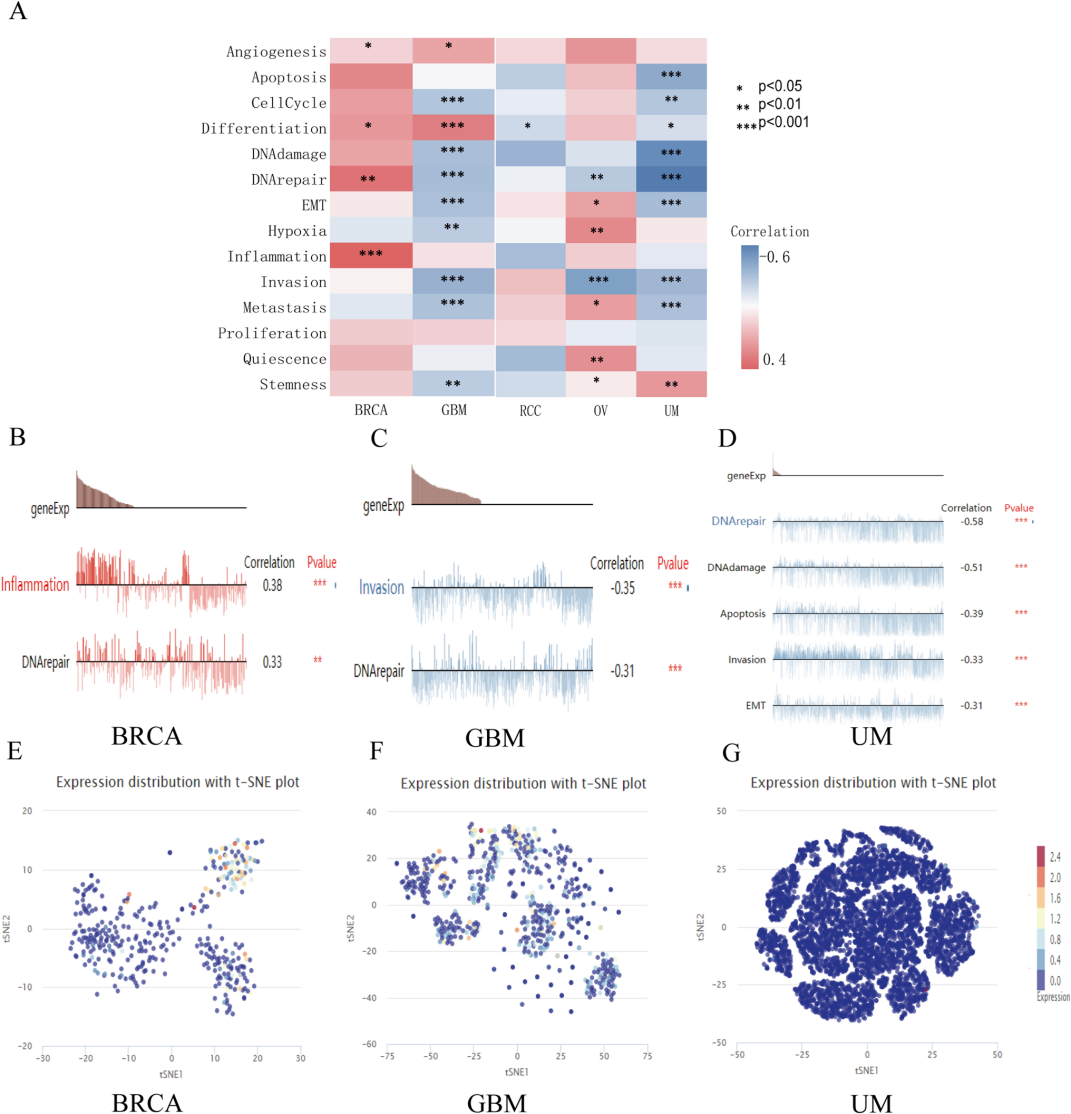
IL4I1 expression and cancer functional states at a single-cell level. **A** IL4I1 expression was correlated with cancer functional states in pan-cancer. **B**–**D** The association between IL4I1 expression and cancer function in BRCA, GBM, and UM. (**E**–**G**) The t-SNE plot indicated IL4I1expression profile in single cells of BRCA, GBM, and UM. (*, p < 0.05, **, p < 0.01, ***, p < 0.001)

The expression profile of IL4I1 at the single-cell level and its potential functional status in pan-cancer were explored through an analysis of CancerSEA. As shown in Fig. 7A, IL4I1 expression was significantly positively correlated with inflammation, angiogenesis, and DNA repair in BRCA, IL4I1 expression was negatively correlated with cell cycle, DNA damage, DNA repair, EMT, invasion, and metastasis in GBM and UM, and IL4I1 expression was positively correlated with EMT, hypoxia, Quiescence, and stemness in OV. Figure 7B shows the relationship between IL4I1 expression and DNA repair, inflammation in BRCA. Figure 7C shows the relationship between IL4I1 expression and DNA repair, invasion in GBM, and Fig. 7D shows the relationship between IL4I1 expression and DNA repair, apoptosis, DNA damage and invasion, EMT in UM. In addition, T-SNE plots showed the distribution of IL4I1 expression in individual cells of BRCA, GBM and UM (Fig. 7E–G). Taken together, these results suggest that IL4I1 may play an important role in tumor progression.

### Single-cell transcriptome analysis of the IL4I1 expression in the glioma tumor microenvironment

**Fig. 8:**
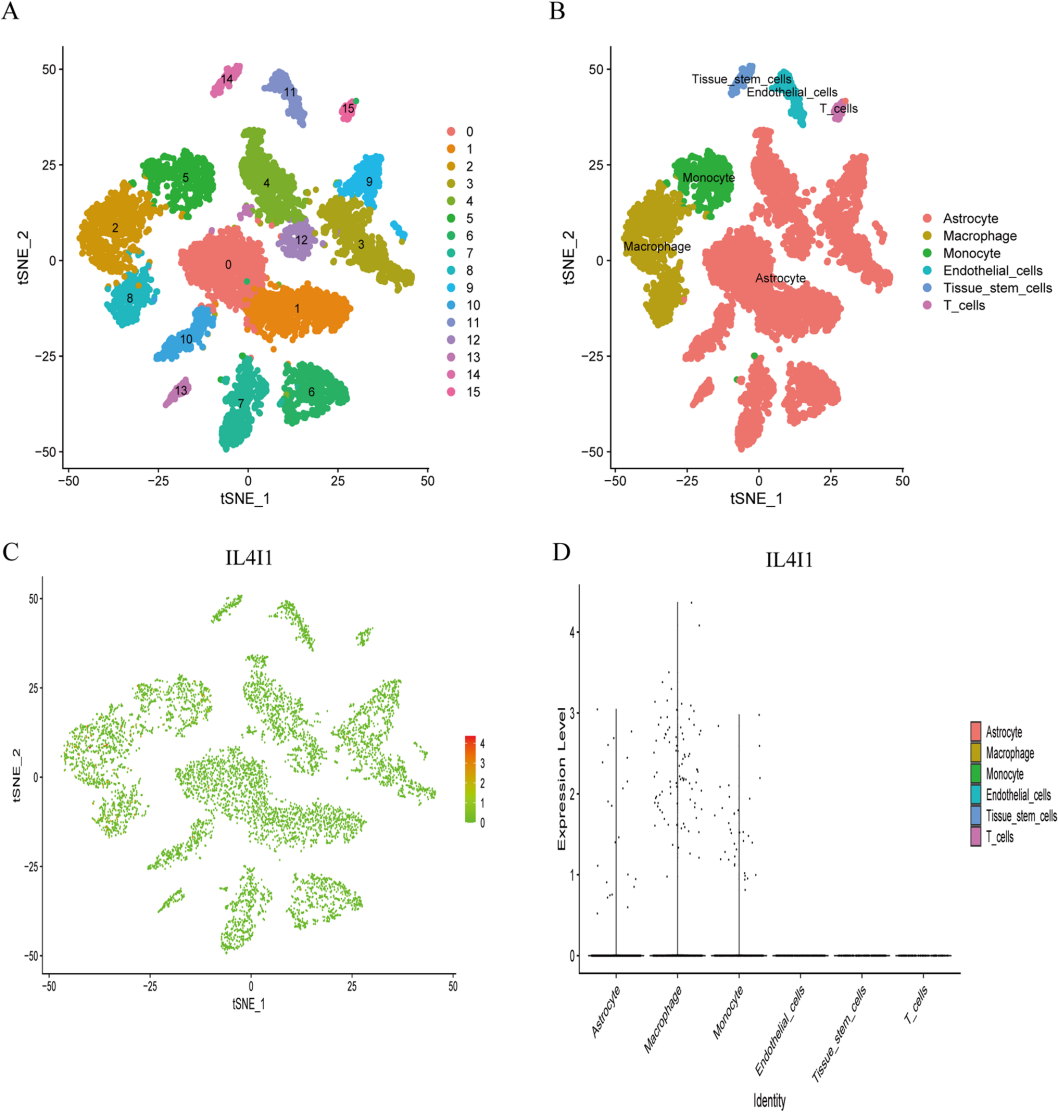
Single-cell transcriptomic atlas of glioma. **A** t-SNE plot colored by various cell clusters. **B** The cell types identified by marker genes. **C** A tSEN plot of IL4I1expression in different cell types. **D** Comparison of IL4I1expression in different glioma tumor microenvironment cells

Four single-cell samples of gliomas were subjected to scRNA-seq analysis (Table 1). After quality control (QC) using Seurat, information from 7919 high-quality single cells was utilized for subsequent analysis. Cell clustering analysis based on the t-Distributed Stochastic Neighbor Embedding (tSNE) algorithm showed that the above cells could be classified into 15 classes (Fig. 8A), and subsequent cell annotation yielded six classes of cells, namely, astrocytes, macrophages, monocytes, endothelial cells, tissue stem cells, and T cells (Fig. 8B). Furthermore, we compared IL4I1 expression in different cell types (Fig. 8C), and we found low expression in the majority of cells and high expression only in macrophages. Similarly, we found significant differences in IL4I1 expression in different cells, with the highest expression in macrophages (Fig. 8D). These results suggest that IL4I1 is significantly different in different cells of the glioma tumor microenvironment and that targeting IL4I1 may be a breakthrough in regulating the tumor microenvironment.

**Table 1:** Sample information

GEO No	Sample	Diagnosis	Gender	Age
GSE103224	GSM2758472	Glioblastoma, WHO grade IV	Male	62y
	GSM2758473	Glioblastoma, WHO grade IV	Male	65y
	GSM2758475	Anaplastic Astrocytoma, WHO grade III	Female	56y
	GSM2758476	Glioblastoma, recurrent	Female	63y

### IL4I1-binding protein network and methylation analysis

**Fig. 9:**
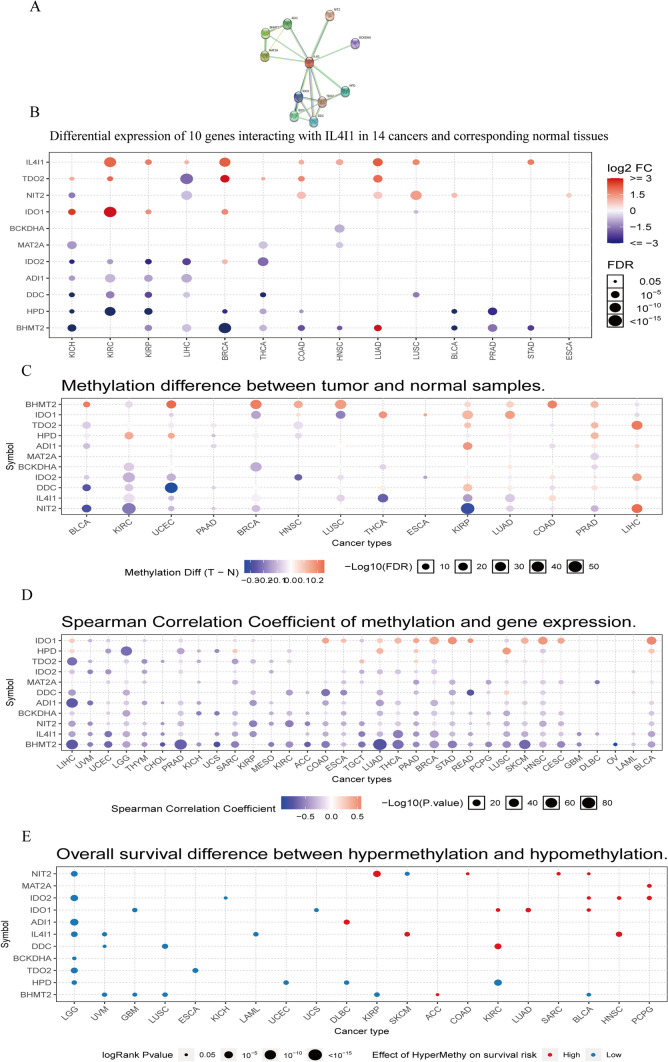
IL4I1-binding protein network and methylation analysis. **A** IL4I1-correlated network. **B** Significant differential mRNA expression of IL4I1 and its binding proteins in normal and tumor tissues in TCGA database. **C** Differential methylation of IL4I1 and its binding proteins between tumor (T) and normal (N) samples in each type of cancer. Red and blue dots represent increased and decreased methylation in tumors, respectively. Higher color intensity indicates larger difference in the level of methylation. **D** Correlation between methylation and mRNA gene expression. Red and blue points represent a positive and negative correlation, respectively. Higher color intensity denotes stronger correlation. **E** The relationship between high and low methylation levels of IL4I1 and its binding proteins and patient survival. Red and blue dots represent worse and better survival of patients in the hypermethylation group, respectively. The dot size represents the statistical significance; larger dots indicate higher statistical significance

The functional enrichment of IL4I1 in cancer was investigated by mapping its protein interaction network using the STRING database. The results revealed 10 genes which constituted the protein interaction network, namely acireductone dioxygenase 1 (ADI1), branched chain keto acid dehydrogenase E1 subunit alpha (BCKDHA), betaine-homocysteine S-methyltransferase 2 (BHMT2), dopa decarboxylase (DDC), 4-hydroxyphenylpyruvate dioxygenase (HPD), indoleamine 2,3-dioxygenase 1 (IDO1), IDO2, methionine adenosyltransferase 2A (MAT2A), nitrilase family member 2 (NIT2), tryptophan 2,3-dioxygenase (TDO2), and Interleukin-4 inducible gene 1 (IL4I1) (Fig. 9A).

Subsequently, the expression of 11 genes in the pan-cancer setting was analyzed using the GSCALite platform. Results indicated high expression of IL4I1, TDO2, NIT2, and IDO1, while IDO2, ADI1, DDC, HPD, BHMT2 exhibited low expression in most cancers (Fig. 9B). Next, we investigated differences in the methylation of IL4I1 and binding proteins between tumor and normal tissues in 14 cancer types. The results showed that IL4I1 methylation was downregulated in most types of cancer, including KIRC, UCEC, BRCA, LUSC, THCA, KIRP, and LUAD (Fig. 9C). Thereafter, we evaluated the correlation between IL4I1 methylation and expression in 32 cancer types. The results showed that the expression levels of IL4I1 and most of its binding proteins were negatively correlated with methylation; the levels of only a few binding proteins were positively correlated with methylation (Fig. 9D). Finally, we evaluated the relationship between the levels of IL4I1 methylation and OS in 22 cancer types. The results showed that hypomethylation of IL4I1 was associated with poor prognosis in LGG, UVM, and LAML, while hypermethylation of IL4I1 was associated with poor prognosis in SKCM and HNSC (Fig. 9E).

### Drug and pathway activity analysis showed the role of IL4I1 in the pan-cancer cohort

**Fig. 10:**
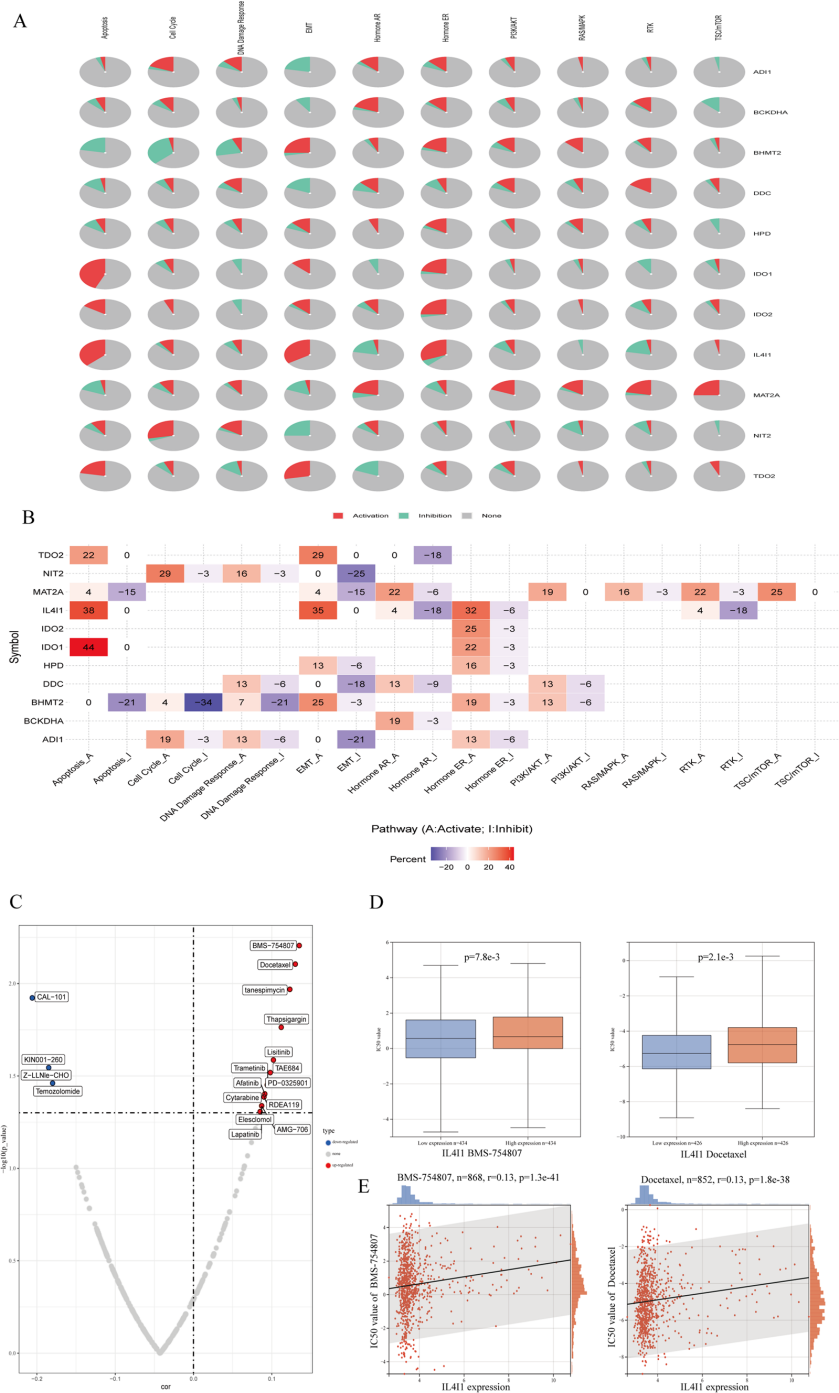
The role of IL4I1 in established cancer-related pathways (GSCALite), and drug analysis of common genes based on GDSC data. **A** Pie chart of pathway activity. **B** Heatmap of pathway activity. **C** Volcano plot of the correlation between IL4I1 and the IC50 values of drugs. Blue and red dots indicate negative and positive correlation groups, respectively. **D** IC50 values of BMS-754807 and docetaxel in high- and low-IL4I1 groups. **E** Correlation between IL4I1 expression and the IC50 values of BMS-754807 and docetaxel

The relationship between the expression of IL4I1-related genes and their protein interactions, and the activation or inhibition of common signaling pathways, was investigated using the GSCALite platform. The results of the pathway activity analysis showed that IL4I1 was related to epithelial–mesenchymal transition (EMT), apoptosis, cell cycle and estrogen receptor activation, and the inhibition of hormone androgen receptor, phosphatidylinositol 3 kinase/protein kinase B (PI3K/AKT), and receptor tyrosine kinase (RTK) pathways (Fig. 10A, B).

We downloaded the IC50 values of anticancer drugs and gene expression profiles of related cell lines from the GDSC database. To explore the effect of IL4I1 expression on the sensitivity to anticancer drugs, we compared the correlation between the expression of IL4I1 and the IC50 of those agents. We screened 18 anticancer drugs; of those, four were negatively correlated with IL4I1, including CAL-101 (PI3K inhibitor), temozolomide, and other anticancer drugs. The remaining 14 drugs were positively correlated with IL4I1, including BMS-754807 (insulin-like growth factor 1 receptor/insulin receptor [IGF-1R/IR] inhibitor), docetaxel, afatinib (tyrosine kinase inhibitor), and trametinib (MEK inhibitor) (Fig. 10C). To investigate the effect of IL4I1 expression on the sensitivity to anticancer drugs, tumor cells were divided into high- and low-IL4I1 groups, and their IC50 values were compared. The IC50 of BMS-754807 and docetaxel were increased in the high-IL4I1 group versus the low-IL4I1 group (Fig. 10D). In addition, the IC50 values of BMS-754807 and docetaxel were positively correlated with the expression of IL4I1 (Fig. 10E). These results suggest that patients with high expression of IL4I1 may be resistant to BMS-754807 and docetaxel.

### Validation of IL4I1 expression in glioma tissues

Our study found that the expression level of IL4I1 in gliomas was significantly higher than that in normal brain tissue (Figure S1A). Next, we performed a survival analysis, and similar to the previous results, the higher the expression level of IL4I1 the shorter the survival time of the patients (Figure S1B, Supplementary Table S1). This suggests that IL4I1 is a potential target for glioma treatment.

## Discussion

IL4I1, a secretory L-phenylalanine oxidase, is expressed by antigen-presenting cells. In vitro, IL4I1 inhibits T cell proliferation and cytokine production. Furthermore, IL4I1 partly promotes the differentiation of immature CD4 + T cells into Treg cells by producing H2O2 and consuming phenylalanine in the T cell microenvironment [23]. IL4I1 is detected in some tumors (e.g., B-cell lymphomas) [24]. Additionally, in human skin melanoma, IL4I1 influences patient survival and prognosis by modulating the local T cell response [25]. The correlation between IL4I1 protein or mRNA and prognosis was also observed in KIRC [7], glioma [26], COAD [27], and BRCA [28]. However, the function of IL4I1 in numerous other types of tumors remains unclear.

Tumor tissue data from TCGA and normal tissue data from TCGA and GTEx databases were initially used to evaluate IL4I1 expression. We found that IL4I1 was highly expressed in 25 types of tumors, namely ACC, bladder urothelial carcinoma, BRCA, CESC, cholangiocarcinoma, COAD, ESCA, GBM, HNSC, KIRC, KIRP, LAML, LGG, LIHC, LUAD, LUSC, OV, PAAD, PRAD, READ, SKCM, STAD, THCA, UCEC, and UCS. IL4I1 was found to be lowly expressed only in KICH. In the CPTAC dataset, IL4I1 total protein was highly expressed in BRCA, COAD, OV, KIRC, HNSC, PAAD, GBM, LIHC, UCEC, and LUAD. The overexpression of IL4I1 in GBM, KIRP, LAML, LGG, LIHC, and UVM, indicates worse OS for patients with tumors. In contrast, in patients with SKCM, the overexpression of IL4I1 indicates better OS for patients with cancer. Previous studies have shown that IL4I1 is highly expressed in gliomas. IL4I1 promotes tumor cell movement and inhibits T cell proliferation; these effects are negatively correlated with the OS rate of patients with glioma [9]. However, the role of IL4I1 in KIRP, LAML, LIHC, and UVM has not been determined.

We observed that the methylation of IL4I1 was reduced in KIRC, UCEC, BRCA, LUSC, THCA, KIRP, and LUAD, and was negatively correlated with the expression of IL4I1. In LGG, UVM and LAML, the hypomethylation of IL4I1 was associated with poor prognosis. In SKCM and HNSC, the hypermethylation of IL4I1 was associated with poor prognosis. Consequently, IL4I1 methylation levels can serve as a prognostic marker.

In the tumor microenvironment, tumor-domesticated immune and stromal cells, including TAM and CAF, play a crucial role in accelerating tumor progression. The remodeling of immune cells by tumor cells can lead to immune escape [29, 30]. In the present study, through GSEA, we predicted that IL4I1 participates in immunomodulatory pathways. In addition, according to the analysis of data from the TIMER2 database, we found that the expression of IL4I1 was positively correlated with TAM and CAF. The results showed that IL4I1 directly or indirectly regulated the levels of TAM and CAF infiltration. Based on these results, we further investigated the relationship between IL4I1 and immune checkpoints, MHC, immunosuppressive genes, chemokines, and chemokine receptors. Chemokine and chemokine receptors (e.g., CCL5, CXCL9, CXCL10, CXCR6, CCR1, and CCR5) were positively correlated with the expression of IL4I1 in most tumors. MHC, immunostimulatory genes, and immunosuppressive genes (e.g. HLA-E, HLA-B, HAVCR2, CSF1R, PDCD1LG2, CD86, CD27, CD28, and TNFRSF9) were positively correlated with the expression of IL4I in most tumors. Furthermore, immune checkpoint genes (e.g., CTLA4, PDCD1, CD274, CD80, and PRF1) were positively correlated with IL4I1 in most tumors. These findings indicate that IL4I1 is closely associated with immune regulation and may serve as a novel immune checkpoint. Notably, patients with tumors expressing high levels of IL4I1 may show a state of immunosuppression.

By investigating the IL4I1 pathway, we observed that IL4I1 is linked to the activation of EMT, apoptosis, cell cycle, and hormone estrogen receptor, and associated with the inhibition of hormone androgen receptor and the PI3K/AKT and RTK pathways. Previous studies reported that cell cycle disorders lead to abnormal cell proliferation, reduction of apoptosis, invasion, and metastasis [31]. Therefore, IL4I1 may promote tumorigenesis by regulating the cell cycle, EMT, apoptosis, and other mechanisms.

As IL4I1 has been identified as a promising new therapeutic target, we sought to investigate the role of IL4I1 in anticancer drug selection. For this purpose, we used data from the GDSC database and analyzed the correlation between the expression of IL4I1 and IC50 values of anticancer drugs. The findings revealed that the IC50 of BMS-754807 and docetaxel were increased in the high-IL4I1 group versus the low-IL4I1 group. In addition, the IC50 values of BMS-754807 and docetaxel were positively correlated with the expression of IL4I1. These results suggest that patients with high expression of IL4I1 may be resistant to treatment with BMS-754807 and docetaxel. Previous studies have shown that BMS-754807, as an inhibitor of IGF1R/IR, may be used in the treatment of gliomas and SKCM [32, 33]. Of note, docetaxel is used in the treatment of PRAD, BRCA, and LUAD [34–36]. There is a negative correlation between temozolomide and IL4I1 expression, and temozolomide is commonly utilized as a chemotherapeutic drug in patients with gliomas [37]. The above evidence provides a new direction in the selection of drugs for the treatment of tumors in the future. Recently several computational approaches have been proposed to rapidly screen drug candidates for drug discovery. For example, ITCM database to screen the active ingredients in herbal medicines against tumours [38] and COIMMR to reveal the contribution of herbal ingredients to human cancers through immune microenvironment and metabolic reprogramming [39]. However, in this paper the active ingredients of herbal medicines were not investigated for corresponding studies on IL4I1, which may be a limitation in our study.

## Conclusion

We conducted a comprehensive evaluation of IL4I1, revealing its potential role as a promoter of cancer and prognostic indicator in patients. Importantly, high expression of IL4I1 typically suggests tumor immunosuppression, which may render immune checkpoint inhibitors unsuitable for treatment. Finally, patients with high levels of IL4I1 expression may be resistant to BMS-754807 and docetaxel, but sensitive to temozolomide. Based on the extensive influence of IL4I1 on the efficacy of anticancer drugs, it is necessary to fully understand its role as a biomarker of patient prognosis. This knowledge could significantly impact the progression and treatment strategies of various cancer types.

### Supplementary Information

**Additional file 1: **(DOCX 84 KB)

**Additional file 2: **(XLSX 11 KB)

## Data Availability

All data are available from TCGA, GTEx and GEO.

## Abbreviations

ACC: Adrenocortical carcinoma
BLCA: Bladder urothelial carcinoma
BRCA: Breast invasive carcinoma
CESC: Cervical squamous cell carcinoma and endocervical adenocarcinoma
CHOL: Cholangiocarcinoma
COAD: Colon adenocarcinoma
DLBC: Lymphoid neoplasm diffuse large B-cell lymphoma
ESCA: Esophageal carcinoma
GBM: Glioblastoma multiforme
HNSC: Head and neck squamous cell carcinoma
KICH: Kidney chromophobe
KIRC: Kidney renal clear cell carcinoma
KIRP: Kidney renal papillary cell carcinoma
LAML: Acute myeloid leukemia
LGG: Brain lower grade glioma
LIHC: Liver hepatocellular carcinoma
LUAD: Lung adenocarcinoma
LUSC: Lung squamous cell carcinoma
MESO: Mesothelioma
NSCLC: Non-small cell lung cancer
OV: Ovarian serous cystadenocarcinoma
PAAD: Pancreatic adenocarcinoma
PCPG: Pheochromocytoma and paraganglioma
PRAD: Prostate adenocarcinoma
READ: Rectum adenocarcinoma
SARC: Sarcoma
SKCM: Skin cutaneous melanoma
STAD: Stomach adenocarcinoma
TGCT: Testicular germ cell tumor
THCA: Thyroid carcinoma
THYM: Thymoma
UCEC: Uterine corpus endometrial carcinoma
UCS: Uterine carcinosarcoma
UVM: Uveal melanoma
