# Supplementary material for: Pan-cancer analysis of prognostic and immunological role of IL4I1 in human tumors: a bulk omics research and single cell sequencing validation
Source: Discov Oncol. 2024 May 1;15:139. doi: 10.1007/s12672-024-01000-5 (PMC11063023; doi:10.1007/s12672-024-01000-5)

Figure S1 Validation of IL4I1 expression in glioma tissues. (A) qRT-PCR was used to detect the relative expression of IL4I1 in glioma tissues (n = 68) and normal brain tissues (n = 15). (B) Kaplan-Meier (KM) survival curve can reveal the correlation between OS and IL4I1.


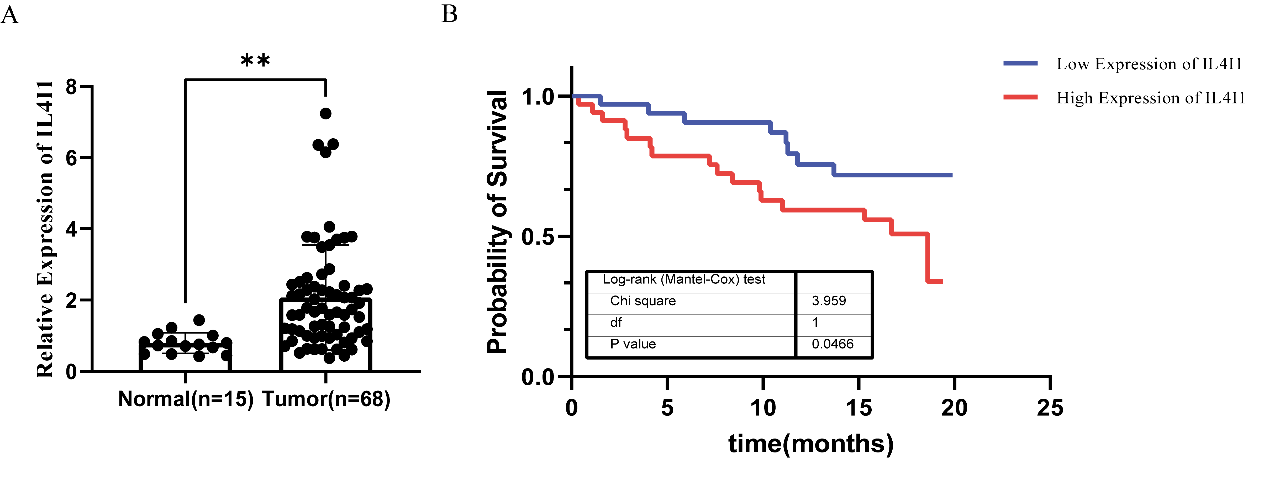

Supplement: Supplementary file 1 — Additional file 1: (DOCX 84 KB) [file 12672_2024_1000_MOESM1_ESM.docx]
